# Income Is a Stronger Predictor of Mortality than Education in a National Sample of US Adults

**DOI:** 10.3329/jhpn.v30i1.11280

**Published:** 2012-03

**Authors:** Charumathi Sabanayagam, Anoop Shankar

**Affiliations:** ^1^Department of Community Medicine, West Virginia University School of Medicine, Morgantown, WV 26506, USA; ^2^Singapore Eye Research Institute, Singapore

**Keywords:** Education, Income, Inequality, Mortality, Risk factors, Socioeconomic conditions, United States

## Abstract

Low socioeconomic status (SES) is associated with mortality in several populations. SES measures, such as education and income, may operate through different pathways. However, the independent effect of each measure mutually adjusting for the effect of other SES measures is not clear. The association between poverty-income ratio (PIR) and education and all-cause mortality among 15,646 adults, aged >20 years, who participated in the Third National Health and Nutrition Examination Survey in the USA, was examined. The lower PIR quartiles and less than high school education were positively associated with all-cause mortality in initial models adjusting for the demographic, lifestyle and clinical risk factors. After additional adjustment for education, the lower PIR quartiles were still significantly associated with all-cause mortality. The multivariable odds ratio (OR) [95% confidence interval (CI)] of all-cause mortality comparing the lowest to the highest quartile of PIR was 2.11 (1.52-2.95, p trend≤0.0001). In contrast, after additional adjustment for income, education was no longer associated with all-cause mortality [multivariable OR (95% CI) of all-cause mortality comparing less than high school to more than high school education was 1.05 (0.85-1.31, p trend=0.57)]. The results suggest that income may be a stronger predictor of mortality than education, and narrowing the income differentials may reduce the health disparities.

## INTRODUCTION

Socioeconomic inequalities in health are a major public-health concern. Epidemiological studies have shown that socioeconomic status (SES) is associated with mortality in several populations (1-10). Although SES measures, such as education and income, are correlated to each other, they may also have an independent effect on mortality (11,12). Thus, studying the role of each SES measure on mortality after mutually adjusting for other SES measures is necessary to understand the independent role of social differentials in mortality. In this context, we examined the independent effect of education and income on mortality by simultaneously adjusting for each other, in addition to the demographic, lifestyle and clinical factors.

## MATERIALS AND METHODS

The Third National Health and Nutrition Examination Survey (NHANES III) collected data on a nationwide probability sample of the civilian non-institutionalized US population. Detailed descriptions of the complex survey design, interviewing procedures, and physical examinations conducted have been published before and are available online (13).

Information on mortality status was available for 18,800 participants aged ≥20 years. We further excluded those with missing data on poverty-income ratio (PIR, n=1,978), education (n=96), and other variables (n=1,080) included in the multivariable model, leaving 15,646 available for current analysis. Compared to those who were included in the final analysis, those who were excluded (n=3,154) were: older, more likely to be female, primary or below educated, to have lower PIR, less likely to be non-Hispanic whites, consume alcohol, physically active, and had higher levels of mean arterial blood pressure (MABP) (all p<0.05).

The main outcome of interest—all-cause mortality—was recorded from the NHANES III-linked mortality file provided by the National Center for Health Statistics (NCHS). The participants were followed up for mortality for up to 12 years from 1988-1994 through 31 December 2006. Ascertainment of mortality was based upon a probabilistic match between the NHANES III and the death certificate records of the National Death Index. Education and PIR were chosen as indicators of SES. PIR was computed as a ratio of the mid-point of the observed family-income category to the family's appropriate poverty threshold set by the US Census Bureau in a given calendar year. The educational status based on completed years of education was categorized into <high school graduate (<12 years), high school graduate (12 years), and >high school graduate (>12 years, including college degree).

We first examined the association between PIR and all-cause mortality and subsequently examined the association between education and all-cause mortality. To examine the independent effect of education and income on all-cause mortality, we used three logistic regression models: (a) the age, sex-adjusted model; (b) the multivariable-adjusted model 1, additionally adjusting for race-ethnicity, marital status, smoking, alcohol intake, physical activity, MABP, body mass index, high-density lipoprotein cholesterol, and (c) the multivariable-adjusted model 2, adjusting for all variables in the multivariable model 1 plus mutual adjustment of other SES measures (education in models of PIR and PIR in models of education). The trends in the odds ratio (OR) of all-cause mortality across the categories of PIR and education were tested by modelling each SES indicator category as an ordinal variable in the corresponding multivariable model. In subgroup analyses, we examined the association among education, income, and all-cause mortality stratified by race-ethnicity and gender using the multivariable model 2. All analyses were weighted to account for unequal probabilities of selection, oversampling, and non-response using the SUDAAN software (version 8.0) (Research Triangle Institute, Research Triangle Park, NC) and the SAS software (version 9.2) (SAS Institute, Cary, NC).

### Ethical approval

Participants signed an informed consent form before interview in the home. The ethical approval was obtained from the Institutional Review Board of the National Center for Health Statistics of the Centers for Disease Control and Prevention.

## RESULTS

Table 1 shows the ORs for mortality in relation to selected factors included in the multivariable model. Age, race-ethnicity, being never married, and former and current smoking were positively associated with all-cause mortality whereas female sex, current drinking, and physical activity were inversely associated with all-cause mortality. Table 2 shows the association among PIR and education and all-cause mortality. The lower PIR quartiles were positively associated with mortality in both age, sex-adjusted model and multivariable model 1 that was additionally adjusted for the demographic, lifestyle and clinical risk factors (p trend <0.0001). Additional adjustment for education in the multivariable model 2, though attenuated this association, it still remained significant (p trend <0.0001). Education was associated with mortality in the age, sex-adjusted model and in the multivariable model 1 (p trend=0.0006). Additional adjustment for income in the multivariable model 2, however, weakened this association considerably, and it was no longer significant (p trend=0.57).

Table 3 shows the association between the SES and all-cause mortality in subgroups of race-ethnicity and gender. In general, the positive association between lower PIR and mortality was consistently present in categories of gender and race-ethnicity. Consistent with the main findings, the association between education and all-cause mortality was not significant in either men or women. When stratified by race-ethnicity, the association between education and all-cause mortality was not significant in non-Hispanic whites but was stronger in other race-ethnicities. However, there was no significant interaction by race-ethnicity (p-interaction=0.3).

## DISCUSSION

In a contemporary, multi-ethnic sample of US adults, we found that the lower PIR quartiles were positively associated with all-cause mortality, independent of the demographic, lifestyle and clinical risk factors. This association was persistent after additional adjustment for education and was consistently present in subgroups of gender and race-ethnicity. In contrast, education was not associated with all-cause mortality after additional adjustment for income.

The majority of the US studies that examined the association between the SES and mortality have used education (5,6) or income (7,8) as measures of SES. The results of our study suggest that income was independently associated with mortality after accounting for the effect of education. In the Americans’ Changing Lives Survey, income differentials in mortality persisted after accounting for education and behavioural factors (7). Similarly, it was shown that income overrides the effect of education and occupation on mortality among a large cohort of insurance enrollees in Germany (14). A study in the USA that assessed insurance as an SES measure reported that lack of insurance was associated with mortality after adjustment for education and income (10). Income provides opportunities for healthy lifestyle, spending power, better housing, and access to medical care (12,15,16). In the present study, education was not associated with mortality after accounting for income, suggesting that the educational differences in mortali-ty may partly be mediated through the differences in income, consistent with previous reports (7,17).

**Table 1.** Odds ratios for mortality in relation to selected factors in multivariable model, including income and education

Characteristics	Multivariable OR (95% CI)	p value
Model significance		
Female	0.59 (0.50-0.69)	<0.0001
Race		
Non-Hispanic whites	1.00 (Reference)	
Non-Hispanic blacks	1.24 (0.95-1.62)	0.1
Mexican Americans	1.04 (0.82-1.32)	0.7
Others	0.41 (0.22-0.75)	0.004
Marital status		
Married/living as married	1.00 (Reference)	
Never married	1.34 (1.02-1.76)	0.03
Others	1.48 (0.99-2.21)	0.05
Smoking categories		
Never	1.00 (Reference)	
Former	1.47 (1.20-1.80)	0.0002
Current	2.25 (1.75–2.91)	<0.0001
Alcohol drinking	0.76 (0.60-0.96)	0.01
Physical activity	0.65 (0.54-0.78)	<0.0001
Age, per unit increase	1.10 (1.09-1.11)	<0.0001
Mean arterial BP, per 10 mm Hg increase	1.01 (1.00-1.01)	0.08
Body mass index (kg/m^2^), per unit increase	1.00 (1.00-1.00)	0.1
HDL-cholesterol (mg/dL), per 10 units decrease	1.01 (0.95-1.08)	0.7

BP=Blood pressure;

CI=Confidence interval;

HDL=High-density lipoprotein;

OR=Odds ratio

**Table 2.** Association between socioeconomic status and mortality

SES measure	No. (n=15,646)	Mortality rate (%)	Age and sex adjusted OR (95% CI)	Multivariable model 1* OR (95% CI)	Multivariable model 2† OR (95% CI)
Poverty-income ratio quartiles					
Quartile 4 (3.4-11.9)	3,943	6.6	1.00 (referent)	1.00 (referent)	1.00 (referent)
Quartile 3 (2.1-3.3)	4,015	8.1	1.14 (0.94-1.39)	1.03 (0.83-1.28)	1.01 (0.81-1.26)
Quartile 2 (1.2-2.0)	3,834	14.9	1.94 (1.56-2.42)	1.75 (1.39-2.21)	1.73 (1.34-2.23)
Quartile 1 (0-1.1)	3,854	15.0	2.86 (2.15-3.82)	2.14 (1.58-2.90)	2.11 (1.52-2.95)
p trend			<0.0001	<0.0001	<0.0001
Education					
>high school	4,579	5.7	1.00 (referent)	1.00 (referent)	1.00 (referent)
High school	4,843	8.8	1.40 (1.15-1.71)	1.23 (1.01-1.50)	1.15 (0.95-1.39)
<high school	6,224	18.0	1.67 (1.37-2.03)	1.32 (1.08-1.61)	1.05 (0.85-1.31)
p trend			<0.0001	0.006	0.57

*Adjusted for age (years), sex (women, men), race-ethnicity (non-Hispanic whites, non-Hispanic blacks, Mexican Americans, others), marital status (married/living as married, never married, others), smoking categories (never, former, current), current drinker (absent, present), mean arterial blood pressure (mm Hg), high-density lipoprotein cholesterol (mg/dL), body mass index (kg/m^2^), and physical activity (absent, present);

†Adjusted for all variables in multivariable model 1 + mutual adjustment of SES variable (education in models of poverty-income ratio and vice-versa);

CI=Confidence interval;

OR=Odds ratio;

SES=Socioeconomic status

**Table 3.** Association between socioeconomic status and mortality stratified by race-ethnicity

SES measure	Multivariable OR (95% CI)*		Multivariable OR (95% CI)*	
	Non-Hispanic whites (n=6,993)	Other race-ethnicities (n=8,653)	Men (n=7,414)	Women (n=8,232)
Poverty-income ratio				
Quartile 4	1.00 (referent)	1.00 (referent)	1.00 (referent)	1.00 (referent)
Quartile 3	0.97 (0.77-1.23)	1.19 (0.71-1.99)	1.17 (0.86-1.60)	0.83 (0.59-1.15)
Quartile 2	1.73 (1.29-2.31)	1.61 (1.09-2.36)	1.97 (1.45-2.68)	1.44 (0.96-2.16)
Quartile 1	2.29 (1.52-3.43)	1.68 (1.05-2.69)	2.69 (1.69-4.28)	1.62 (1.10-2.40)
p trend	<0.0001	0.03	<0.0001	0.009
Education				
>high school	1.00 (referent)	1.00 (referent)	1.00 (referent)	1.00 (referent)
high school	1.12 (0.89-1.41)	1.32 (0.88-1.98)	1.29 (1.05-1.60)	1.03 (0.76-1.40))
<high school	0.96 (0.74-1.24)	1.66 (1.03-2.68)	0.95 (0.70-1.27)	1.16 (0.86-1.57)
p trend	0.71	0.03	0.66	0.30

*Adjusted for age (years), marital status (married/living as married, never married, others), smoking categories (never, former, current), current drinker (absent, present), mean arterial blood pressure (mm Hg), high-density lipoprotein cholesterol (mg/dL), body mass index (kg/m^2^), physical activity (absent, present), and poverty-income ratio in models of education and vice-versa;

CI=Confidence interval;

OR=Odds ratio;

SES=Socioeconomic status

### Limitations

Although the strengths of the study include its large sample-size, rigorous methodology, and rich information on covariates, our study has some limitations. First, since the SES variables were assessed only once as part of the NHANES III, the effect of changes in income over time on mortality could not be studied. Second, the possibility of residual confounding due to measurement error resulting from broad categorization of covariates, for example smoking and drinking, cannot be excluded.

### Conclusions

In a nationally-representative sample of US adults, we found that lower income was positively associated with mortality, independent of the demographic, lifestyle and clinical risk factors, and this association persisted after additional adjustment for education. In contrast, after additional adjustment for income, lower education was not associated with mortality. Our results suggest that income may be a stronger predictor of mortality than education, and narrowing the income differentials may reduce health disparities.

## ACKNOWLEDGEMENTS

The study was funded by an American Heart Association National Clinical Research program grant (AS).
